# Novel *KIT* mutation, D816_N819delinsll, in a patient with systemic mastocytosis: a case report

**DOI:** 10.1007/s00428-025-04237-9

**Published:** 2025-09-02

**Authors:** Hazem A. Juratli, Hanna Wassmer, Darius Juskevicius, Ilaria Alborelli, Karin Hartmann, Alexandar Tzankov

**Affiliations:** 1 Pathology, Institute of Medical Genetics and Pathology, University Hospital Basel and University of Basel, Basel, Switzerland; 2 Histological Diagnostics, Kempf and Pfaltz, Zurich, Switzerland; 3 Department of Dermatology, Division of Allergy, University Hospital Basel and University of Basel, Schoenbeisntrasse 40, 4031 Basel, Switzerland; 4 Diagnostic Hematology Unit, Division of Hematology, University Hospital Basel and University of Basel, Basel, Switzerland; 5 Department of Clinical Research, University Hospital Basel and University of Basel, Basel, Switzerland; 6 Department of Biomedicine, University Hospital Basel and University of Basel, Basel, Switzerland

**Keywords:** Mastocytosis, Systemic mastocytosis, *KIT* mutation, *KIT* variant, *KIT* D816_N819delinsll, Genetic testing

## Abstract

Mast cell (MC) disorders result from inappropriate release of mediators and/or excessive accumulation of MCs, leading to symptoms of various organs and systems. Clonal MC disorders are defined by the presence of phenotypically aberrant and/or *KIT-*mutated MCs, and if aggregates of MCs are detectable, are designated as mastocytosis. Systemic mastocytosis (SM) affects mainly the bone marrow, with or without skin involvement. It is associated with the activating mutation D816V in the *KIT* gene. Other activating *KIT* gene variants are also observable in SM; activating *KIT* mutations are recognized as a minor diagnostic SM-criterion. We report a novel *KIT* variant in a patient with indolent SM, an in-frame deletion-insertion affecting amino acids D816 to N819 (D816_N819delinsll), creating an aliphatic pouch similar to that resulting from the D816V mutation, and leading to MC activation as suggested by the symptoms of the patient and the positivity for phosphorylated STAT5 in the clonal MCs.

## Introduction

Mast cells (MCs) are innate immune cells derived from the myeloid lineage. They originate from CD34 +/KIT + pluripotent hematopoietic progenitors in the bone marrow (BM), differentiate in the BM, and subsequently circulate and undergo terminal maturation in peripheral tissues and organs. This process is strongly influenced by the stem cell factor (SCF), locally produced in the BM and by many tissue cells, which induces the differentiation of immature MCs into mature MCs, the latter being resident in respective tissues and normally absent from peripheral blood [1]. MCs have been associated with several morbid conditions including anaphylaxis, urticaria, and mastocytosis [1].

Mastocytosis is defined as an abnormal clonal accumulation of MCs in various tissues, predominantly the skin and the BM, but also the gastrointestinal tract, the liver, the spleen, and lymph nodes, and virtually any organ can be affected by mastocytosis [1]. The current World Health Organization (WHO-5) [2] and the International Consensus Classification (2022) [3] classify mastocytosis as follows:

1. Cutaneous mastocytosis (CM), a form that only affects the skin, most commonly observable in children [1–4].
2. Systemic mastocytosis (SM), involving at least one extracutaneous organ system, mostly the BM, with or without evidence of cutaneous lesions, and most prevalent in adults [1–4].
3. MC sarcoma, an exceedingly rare and overly aggressive MC neoplasm [5].

The pathogenesis of mastocytosis is tightly linked to constitutive activation of the SCF receptor KIT, primarily driven by a characteristic exon 17 D816V mutation of the *KIT* gene, along with a variety of other rare KIT-activating mutations. This highlights the critical physiological role that KIT plays in MC proliferation and differentiation [1–4]. The detection of such activating mutations is a minor diagnostic criterion in SM [2, 3], and in *KIT* D816V-negative cases, the application of methods covering the entire coding sequence of *KIT* is recommended [1, 4, 6], as impressively illustrated by the case reported here.

## Case presentation and pathological findings

A 37-year-old Swiss male presented with a history of recurrent allergic reactions to celery with skin rash, dizziness, and shortness of breath. Laboratory tests revealed elevated serum tryptase (24.8 ng/ml). No cutaneous lesions indicative of mastocytosis were observed. His medical history revealed a previous episode of wasp sting-induced anaphylaxis, which prompted the initiation of venom immunotherapy and the instruction to carry an epinephrine autoinjector. The persistence of symptoms, including fatigue and excessive sweating, and an episode of loss of consciousness after exposure to pethidine prompted further investigation, including BM biopsy.

**Fig. 1:**
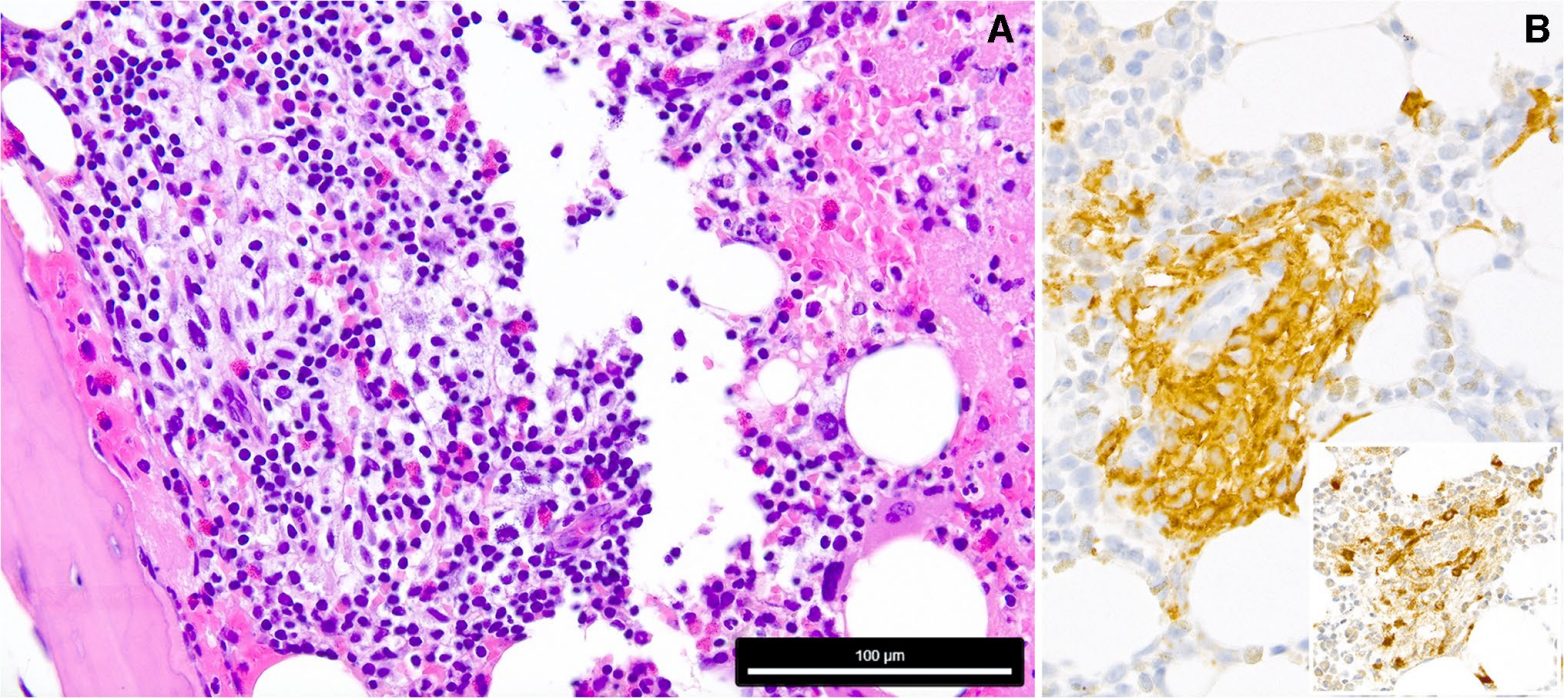
Histological and immunohistochemical analysis of the bone marrow biopsy. **A** Hematoxylin and eosin staining showing a peritrabecular aggregate of epithelioid to spindle-shaped, vaguely granulated mast cells surrounded by eosinophilic granulocytes and lymphocytes. **B** Immunohistochemical staining for CD117 (KIT), highlighting a perivascular mast cell aggregate. Inset: pathologic co-expression of CD25 on the same mast cells. Magnification, × 400

**Fig. 2:**
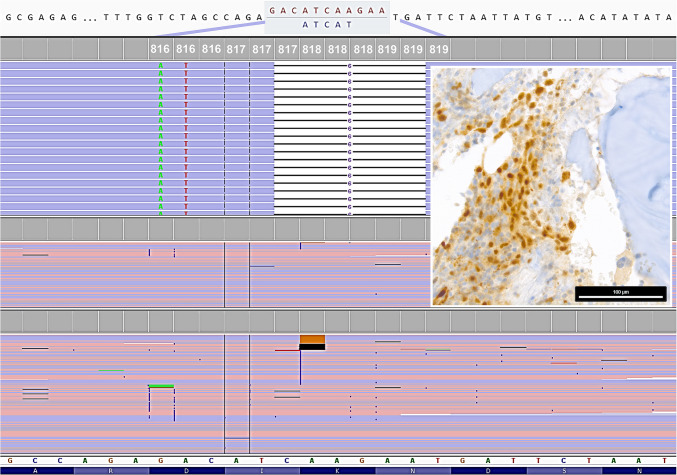
Genomic sequencing data. The NGS alignment displays the sequencing reads mapped to the hotspot reference sequence of the *KIT* gene. The predicted consequence of the mutation at the protein level is the substitution of the crucial aspartic acid (Asp = D) at codon 816 with isoleucine (Ile = I), accompanied by the loss of lysine (Lys = L) and asparagine (Asn = N) at codons 818 and 819, resulting in a KIT protein shortening by two amino acids. Top line blue: detected sequence of nucleotides of neocodons 816 to 817 (ATC-ATT) that encode an Ile-Ile protein sequence; parts of codon 817 and 819, and the whole codon 818 are deleted. The highlighted mutation corresponds to a deletion-insertion event (c.2446_2456delinsATCAT, p.Asp816_Asn819delinsIleIle), visually confirmed by differences in the read alignment. The novel mutation inserts Ile instead of Lys and Asn, which may affect receptor conformation and function. Bottom line: Sequence of amino acids of codons, i.e., Asp-Ile-Lys-Asn for codons 816 to 819. Next bottom line: Reference sequence of nucleotides, i.e., GAC-ATC-AAG-AAT. Inset: Nuclear positivity of phosphorylated STAT5 of the mast cells in situ as a readout of KIT activation by the *KIT* Asp816_Asn819delinsIleIle mutation in the reported patient

The biopsy demonstrated characteristic 3–5% infiltration by CD2 +, CD25 +, CD30 −, CD117 +, and tryptase-positive MCs forming aggregates (Fig. 1). Initial genetic analysis of the biopsy using TaqMan digital droplet (dd) PCR failed to detect the *KIT* D816V mutation. Therefore, an additional NGS-based assay covering all known *KIT* mutational hotspots was performed (Oncomine Focus Assay from Thermo Fisher). Using automated variant detection, no *KIT* mutation could be identified. However, comprehensive manual review of the BAM files uncovered a novel *KIT* mutation: NM_000222.3:c.2446_2456delinsATCAT, p.(D816_N819delinsll) (Fig. 2), with a variant allele frequency (VAF) of 1% in the BM. Upon specific genetic analysis of a later-collected peripheral blood sample, the mutation was also detectable there, with a VAF of 0.07%. Based on these findings, the diagnosis of indolent SM, specifically bone marrow mastocytosis (BMM), was established. To estimate the KIT-activating potential of the D816_N819delinsll *KIT* mutation (beyond its ability to activate MCs as suggested by the clinical symptoms), the BM biopsy was also stained for phosphorylated STAT5 [7], which is considered a good readout for the constitutive activation of the KIT tyrosine kinase in the context of SM [8]. As expected, the MC aggregates of our patient turned out to be highly positive (Fig. 2, insert).

After establishing the diagnosis of BMM, the patient was symptomatically treated with antihistamines. Considering his anaphylaxis history, he was re-advised to carry an epinephrine autoinjector. A regular long-term follow-up strategy to assess disease stability and possible imminent progression was set up, including monitoring of serum tryptase, complete blood counts, and clinical evaluation.

## Discussion

KIT/CD117 is a type III tyrosine kinase (TK) transmembrane receptor encoded by *KIT* on chromosome 4q12. It is a glycoprotein of 145 kDa consisting of 976 amino acids encoded by 21 exons. The protein is divided into extracellular domains, which are characterized by five immunoglobulin-like domains, a transmembrane region, and an intracellular tail that includes a juxtamembrane domain and a TK domain [9, 10].

**Fig. 3:**
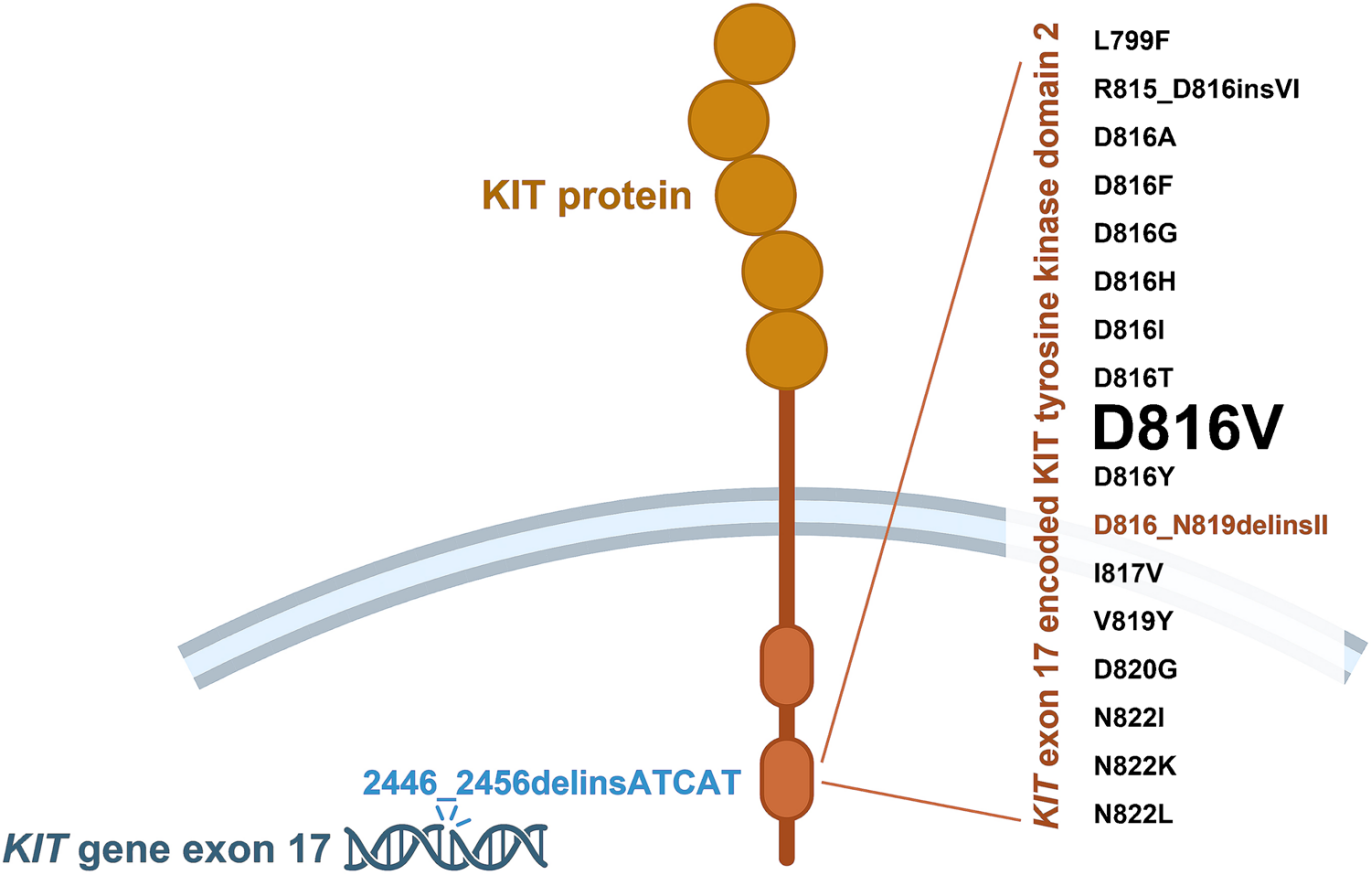
Schematic of the KIT protein structure, illustrating the localization of documented exon 17 mutations of the *KIT* gene in mastocytosis. The novel D816_N819delinsII reported here is in bright brown and its coding sequence change in aquamarine, while the most common D816V mutation is represented in larger font. Created by BioRender and PowerPoint

The *KIT* D816V mutation is frequently detected in SM, with a prevalence exceeding 90% [1]. *KIT* D816V is known to activate downstream pathways, including NF-κB, STAT5, PI3K, mTORC2, and PKC, which are also related to increased activation and accumulation of MCs [10, 11]. Other less common mutations at codon 816 have also been identified in mastocytosis, including D816A, D816F, D816G, D816H, D816I, D816T, and D816Y. Other mutations in nearby codons such as L799F, I817V, V819Y, D820G, N822I, N822K, N822L, R815_D816insVI, E839K, S840N, and S849I (Fig. 3), as well as in more proximal codons (F522C and V560G), that also lead to activation of KIT have been documented in SM too [1, 6, 11, 12]. Thus, the presence of any activating *KIT* mutation is a minor criterion for the diagnosis of SM, and *KIT* mutational testing should be standard in all patients with suspected SM. The detection rate of the most common *KIT* mutation, D816V, varies depending on the sensitivity of the method used, ranging from 30 to 95% [13]. While there is no gold standard detection method, a consensus exists that the sensitivity of the assay should be able to detect VAFs of 0.01%. This level of sensitivity can be achieved using ddPCR or allele-specific oligonucleotide PCR [14].

Given that other activating *KIT* mutations can also occur in SM, as nicely illustrated by our case, diagnostic efforts must continue in instances where *KIT* D816V is not found, but clinical, laboratory, and/or histopathological suspicion of SM remains high. The manual recognition of the previously undescribed D816_N819delinsll variant, which remained initially undetected by the automated variant caller algorithm, highlights the importance of awareness in analyzing NGS data and points to the need for fine-tuning automated variant calling algorithms to achieve reliable mutation detection at lower VAF frequencies. It also demonstrates, in general, the efficacy of NGS in generating appropriate data for diagnostics [15].

The here detected *KIT* mutation is a complex deletion-insertion that affects exon 17 at a known oncogenic hotspot, which is often associated with ligand-independent receptor activation and uncontrolled MC activation and proliferation, especially if the negatively loaded amino acid aspartate (D) is replaced by hydrophobic amino acids such as valine (V), phenylalanine (F), tyrosine (Y), glycine (G), or isoleucine (I) as in D816V/F/Y/G/I [1]. Together with the clinical evidence of MC activation in our patient and the overexpression of phosphorylated STAT5 in his MC in situ, that reflects the constitutive KIT TK activation, we integratively consider the here described *KIT* D816_N819delinsll variant to represent a yet undescribed mutation that should be included in the growing list of activating *KIT* mutations in SM.

## Conclusion

Our case highlights the importance of exhaustive molecular genetic testing in patients with suspected SM in whom conventional tests and algorithms display shortages in identifying oncogenic variants. Our results re-emphasize the implications of *KIT* mutations for diagnostic comprehensiveness and potential therapeutic strategies (e.g. beyond D816V-specific TK inhibition) and point out the necessity of further studies to elucidate the clinical impact of rare *KIT* variants [1, 6]. Practically, our report expands the genetic landscape of SM [1] by an additional novel pathogenic variant, *KIT* D816_N819delinsll.
